# HIV and Ebola: similarities and differences

**DOI:** 10.7448/IAS.17.1.19896

**Published:** 2014-12-01

**Authors:** Mark A Wainberg, Susan Kippax, Marlene Bras, Papa S Sow

**Affiliations:** 1 McGill University AIDS Centre, Jewish General Hospital, Montreal, Quebec, Canada; 2 Journal of the International AIDS Society, Geneva, Switzerland;; 3 Social Policy Research Centre, University of New South Wales, Sydney, Australia; 4 Department of Infectious Diseases, University of Dakar, Dakar, Senegal

The past year has seen the emergence of a new and terrible crisis in world health. Notably, the dramatic spread of the Ebola virus in numerous countries in Western Africa has wreaked havoc throughout that region and brought panic and fear to millions of people around the world. There are, in fact, numerous parallels between people's attitudes towards Ebola virus and its victims today that remind us of attitudes that were prevalent 25 years ago with regard to HIV. In the days prior to the World AIDS Day, December 1, it behoves us to reflect on parallels between HIV and Ebola virus and what the potential impact of the Ebola crisis may be on HIV disease and efforts to control the spread of HIV.

More than 39 million people have died of HIV disease and it is estimated that 35 million individuals are currently infected by the virus [1]. Thus, HIV continues to be a very lethal illness. The number of people infected until now by Ebola is estimated to be approximately 15,300. Of this number, a tragically high proportion has died from their infections, possibly as many as 5400 [2]. This obviously provides testimony to the terrible consequences of contracting the Ebola virus, especially under conditions in which appropriate health care is not available. It is noteworthy that only one health care volunteer from developed countries who contracted Ebola while working in Western Africa has died since returning home. This is largely due to the outstanding treatment they received at tertiary care hospitals in the United States and in Europe following transportation back to their home countries. Hopefully, this may also mean that Ebola is not as transmissible as was feared when good health measures are in place.

We all hope that the Ebola epidemic will soon be contained and that the numbers of new infections will quickly dwindle, as a result of appropriate quarantine and other public health measures. In the meantime, we should remember that approximately ten times more people probably die each day in Africa of HIV/AIDS compared with Ebola.

There is no doubt that Ebola will probably have a major impact on HIV/AIDS, particularly in the countries that have been hit the hardest by the Ebola crisis. First, a disproportionate percentage of those affected and infected by Ebola have themselves been health care workers [3]. This will have negative consequences for the care of persons infected by HIV and other infectious diseases. Moreover, the standard of HIV care in these countries has been below international standards, thus making an already bad situation even worse. Second, the focus of attention has shifted in parts of West Africa to Ebola; this may affect established HIV control measures including HIV testing, treatment of both adults and children with HIV and prevention of mother-to-child transmission of HIV. In addition, widespread fear of Ebola and limitations of public transportation may now make access to HIV clinics more difficult. All of the above are part of the problem of limited human resources. Although at present, numerous wealthy countries around the world contribute billions of dollars annually towards global assistance programmes aimed at procuring lifesaving HIV drugs, many economists have observed that the growing number of HIV-positive persons throughout the world may doom current assistance programmes if they become non-sustainable over the next several decades for reasons of cost.

There are, as mentioned above, a number of parallels between HIV and Ebola with regard to public perceptions of these two dangerous enemies to public health. For one thing, the panic that Ebola has brought to multiple centres through Western Africa, as well as to the United States and Europe, following the return of Ebola-infected individuals, is reminiscent of attitudes towards HIV during the first years of the epidemic. It will be remembered that people of Haitian origin were thought to be a key group of HIV-positive individuals during the early 1980s [4]. For this reason, many people at that time refused to enter taxicabs that were driven by individuals who appeared to be of Haitian descent. This was clearly discriminatory and was not based on scientific evidence. Nonetheless, many thousands of persons were made to feel like second-class citizens and were forced to endure terrible discrimination at that time.

The stigmatization that is now affecting individuals who have volunteered in the most courageous way imaginable to provide care for Ebola-infected individuals in Western African countries also reminds us of the stigma that was attached to individuals who worked with HIV or who cared for individuals who were HIV-positive [5]. The Ebola volunteers clearly deserve our gratitude and are certainly underserving of being stigmatized. Yet, in the early 1980s, few understood that HIV was primarily a sexually transmitted disease and widespread fear emerged that HIV might be casually transmitted among individuals and that contact with health care professionals and scientists working with the virus might lead to HIV transmission. The fact that such fears were groundless did not prevent the emergence of unfounded public attitudes. The result was often refusal on the part of many to come into contact with HIV-positive individuals. In particular, widespread fear and discrimination with regard to gay and other homosexually active men quickly emerged as it was in this population in the United States that AIDS was first identified.

Of course, the intervening decades have seen major progress with regard to HIV disease, to the point that antiretroviral drugs (ARVs) now provide hope to millions of infected individuals around the world to have fully productive lives over indefinite periods. However, this progress has required many years of basic biomedical, clinical and social research. In addition, there has been dramatic improvement in the quality of the ARVs that are used in therapy, in terms of efficacy, tolerability and relative non-occurrence of drug resistance. In contrast, very little comparable research has taken place with regard to Ebola virus, even though this agent was in fact identified prior to the discovery of HIV in 1983. Thus, research into Ebola has badly lagged with regard to prevention efforts, drug discovery and an understanding of patterns of Ebola virus transmission. Obviously, this situation can only be remedied by providing the financial support necessary for this work as well as by providing social support for individuals infected with and affected by Ebola. Indeed, such research in the case of HIV helped tremendously to mitigate problems of HIV discrimination and stigmatization.

The lessons learned from the HIV epidemic in terms of community engagement [6,7] need to be applied immediately to Ebola. Implementation of community awareness sessions and teaching programmes will be necessary to achieve the behavioural and socio-cultural changes that can lead to non-stigmatization and non-discrimination of Ebola-exposed individuals. It needs to be understood that such changes will be an integral component of programmes that will limit the transmission of the Ebola virus in the first place. Furthermore, both the Ebola and HIV epidemics highlight the need to strengthen health care systems in resource-limited settings.

Thus, lessons learned from the HIV epidemic can and should be applied as quickly as possible to the problem of Ebola in order to help end the latter epidemic as quickly as possible. In view of the fact that Ebola causes an acute disease that often leads to death rather than a chronic illness that can be transmitted over many years, it should theoretically be possible to end the Ebola epidemic faster and more efficiently than has been the case for HIV.

Clearly, the only truly successful way of dealing with the HIV epidemic will be to find a cure for HIV infection. Unfortunately, this goal remains elusive in spite of the fact that large numbers of scientists from around the globe are currently engaged in such research as well as on efforts to find a safe and effective HIV vaccine. Hopefully, new research will also quickly lead to the discovery of drugs for Ebola and to the development of a vaccine that will help end the Ebola epidemic as swiftly as it has developed.

## References

[1] Joint United Programme on HIV/AIDS (UNAIDS) (2012). UNAIDS report on the global AIDS epidemic [Internet]. http://www.unaids.org/en/resources/publications/2012/name,76121,en.asp.

[2] Center for Disease Control and Prevention (CDC) (2014). Ebola outbreak in West Africa [Internet]. http://www.cdc.gov/vhf/ebola/outbreaks/2014-west-africa/index.html.

[3] World Health Organization (WHO) (2014). Unprecedented number of medical staff infected with Ebola [Internet]. http://www.who.int/mediacentre/news/ebola/25-august-2014/en/.

[4] Altman LK (1983). Debate grows on U.S. listing of Haitians in AIDS category. *New York Times* [Internet].

[5] Parker R, Aggleton P (2003). HIV and AIDS-related stigma and discrimination: a conceptual framework and implications for action. Soc Sci Med.

[6] Altman D (1994). Power and community: organizational and cultural responses to AIDS.

[7] Parker R (2011). Grassroots activism, civil society mobilization, and the politics of the global HIV/AIDS epidemic. Brown J World Aff.

